# Reply to: Technical challenges of studying the impact of plasma components on the efficacy of lipid nanoparticles for vaccine and therapeutic applications

**DOI:** 10.1038/s41467-024-47726-2

**Published:** 2024-05-09

**Authors:** Kai Liu, Elisa Lázaro-Ibáñez, Michael Lerche, Daniel Lindén, Anna Salvati, Alan Sabirsh

**Affiliations:** 1 Advanced Drug Delivery, Pharmaceutical Sciences, R&D, AstraZeneca, Gothenburg, Sweden; 2 Bioscience Metabolism, Research and Early Development, Cardiovascular, Renal and Metabolism, BioPharmaceuticals R&D, AstraZeneca, Gothenburg, Sweden; 3 Division of Endocrinology, Department of Neuroscience and Physiology, Sahlgrenska Academy, University of Gothenburg, Gothenburg, Sweden; 4 Department of Nanomedicine & Drug Targeting, Groningen Research Institute of Pharmacy, University of Groningen, Groningen, 9713AV The Netherlands

**Keywords:** Nanoparticles, High-throughput screening, Drug delivery

**replying to** J. B. Simonsen *Nature Communications* 10.1038/s41467-024-47724-4 (2024)

The purpose of our publication “Multiomics analysis of naturally efficacious lipid nanoparticle coronas reveals high-density lipoprotein is necessary for their function”^1^ was to elucidate why plasma from obese rats seemed to improve the function of therapeutic lipid nanoparticles (LNP), and use this knowledge to suggest how LNPs might be improved. A comprehensive proteomic analysis revealed that there is a correlation between LNP coronal content, from different individual animals and, LNP function in those same individuals. More effective LNPs tended to have coronal proteomic signatures consistent with increased HDL content. We validated this by creating HDL-augmented coronas artificially and found that these do indeed lead to improved LNP function both in vitro and in vivo. Finally, we conclude that measuring LNP association with certain coronal elements, notably with the HDL component ApoM, is a good predictor for LNP efficacy.

The Matters Arising from Simonsen questions whether the pull-down method we proposed “avoids the co-isolation of lipoproteins and EVs”. We are fully aware of potential lipoprotein and EV contamination in existing LNP corona isolation methods and this was discussed in the original publication^1^. We included multiple controls and validation experiments to ensure that any eventual contaminants do not affect our conclusions. We believe that these efforts contribute significantly to advancing our understanding of LNP coronas and other nanosized particles in complex biological systems.

Several techniques are available to isolate LNPs from biofluids. Density centrifugation, size exclusion chromatography, flow fractionation and pull-down assays may be used. Unfortunately, most of these are relatively slow, require large sample volumes, produce variable results or, they are difficult to parallelize. Systematic exploration of the relationship between LNP composition, LNP coronal content and biofluids from individual animals requires high throughput and speed, good reproducibility and, compatibility with laboratory automation systems. We found that only the pull-down method met these criteria, allowing us to make the large-scale comparisons necessary to see patterns, and to do this quickly enough that the composition of the fragile, metastable coronas can be preserved. High-throughput single-particle sorting would have been ideal, but this method does not currently exist.

LNPs and endogenous particles, like extracellular vesicles (EVs) and lipoproteins, have overlapping physical and chemical characteristics, making separation challenging, so multiple methods and approaches are necessary for analytical evaluation. Rather than using methods which have been optimized for EV isolation as Simonsen suggests, we made use of the fact that LNPs are manufactured and therefore have components, such as PEGylated lipids, that are not normally found in biological systems, allowing us to develop a method for LNP isolation with minimal contamination from similar biological particles that vastly outnumber the LNPs. We believe this is a better approach than trying to optimize methods originally developed for quite different particles such as EVs or, by changing the LNP formulation to include magnetic cores, for example.

Dr. Simonsen’s feedback regarding our publication is also valued and we discuss the points raised below. Readers should also note that while Dr. Simonsen has suggested a number of additional experiments, this is outside the scope of the Matters Arising discourse.

## LNPs and PEG lipid dynamics

We employed antibodies against the polyethylene glycol (PEG) molecules present on the LNP surfaces. These PEGs are “anchored” into the LNPs using acyl chains. Simonsen states that PEG molecules from LNPs can transfer to endogenous nanoparticles, while the two publications he co-authored and cited^2,3^ do not mention PEG or LNPs and thus contain no direct experimental evidence to support his statement. Previous work by us indicated that when LNPs are exposed to biofluids, PEG molecules can “shed” from the LNPs, which is a necessary prerequisite for forming LNP coronas^4^. This work also demonstrated that this process reaches an equilibrium after four hours with about 40% of the PEGylated lipid remaining on the surface of the LNPs. PEG can associate with endogenous nanoparticles and PEG-induced precipitation is sometimes used to purify HDL and EV particles, for example, but the pharmacokinetics of PEG association with endogenous particles within blood or plasma is not well characterized. As previously stated, this is why our study was designed with a validation step that involved creating artificial coronas and testing their effect on LNP function in vitro and in vivo.

Another concern raised by Simonsen is the decreased LNP recovery when extending the Dynabead incubation period from 20 min to 30 min, as shown in Fig. S10a–d^1^ (from the original publication, same below). It is important to clarify that in our experimental setup, all samples tested were incubated, prior to the addition of Dynabeads, with plasma solutions for 4 h (240 min) at 37 °C to allow the PEG shedding to equilibrate and corona to from ref. ^4^. Dynabeads were then added to these pre-incubated samples. A systematic design of experiments methodology was then employed to identify assay parameter values that resulted in the highest LNP recovery with the shortest incubation time, and 20 min was found to be optimal. This optimization used high-throughput formats and automation to fine-tune various inter-related parameters, including, together with the bead incubation times, the epitope detected, the washing buffer composition, the elution pH, elution time and washing steps. The observed reduction in recovery at 30 min could have multiple causes, such as stronger LNP binding to the beads over time or structural collapse of the LNPs. Elucidating the specific cause of this decline will, however, not yield data that influences our conclusions because we validate them conceptually in several ways. In-depth analysis of the material collected using this optimized methodology revealed 1) a complete absence of any EV-associated proteins in the retrieved corona samples, 2) the ability of the recovered particles to transfect cells, 3) that LNP function could be enhanced in vivo by creating artificial coronas based on our observations and, 4) that distinct corona compositions were observed for different LNPs; this diversity in corona composition would be unlikely if extensive contamination were a significant issue during the isolation procedure. Overall, the difference observed in recovery when changing the Dynabead incubation time was just part of the assay optimization and does not invalidate our conclusions.

## Contamination of pull-down samples by endogenous nanoparticles

We expect that LNPs are complexing with specific endogenous nanoparticles within the plasma. Furthermore, we propose that this is necessary for LNP function and the association is driven in part by the LNP composition, as can be seen in both Figs. 5d and 6b^1^. Interestingly, this runs both ways, so if PEG molecules released from LNPs happen to associate with endogenous biomolecules, and then the biomolecules in turn associate with LNPs, we will pull-down LNPs as a “contaminant”, but this is also a valid interaction. The exception to this is if LNP PEG molecules transfer to plasma components and these are then pulled down without any functional LNP components. We did not find that adding PEG lipids to plasma samples could drive effective pull-down of plasma components and, even when PEG was added directly into plasma there was still a significant enrichment of the PEGylated LNPs. This enrichment is shown in Fig. S10e^1^ where primary antibodies against PEG and Cy5 were used, followed by secondary antibodies with near-infrared fluorophores. It is, however, important that this situation is not entirely analogous to PEG lipids supplied by LNPs and, that signals can be caused by individual PEG molecules or PEG micelles, in addition to plasma components that have associated PEG molecules. It is also challenging to compare the anti-PEG and anti-Cy5 (LNP) spots because the antibodies used are all different and this was not designed as a quantitative experiment. Concordantly we do not believe eventual PEG transfer labeling of endogenous plasma components is a problem because following anti-PEG pull-down we can detect LNP lipids using mass spectrometry, we can detect the fluorescent mRNA cargo (using Cy5 label), and the resulting complexes retain LNP transfection capabilities. LNPs with different components and compositions also create different coronal signatures, which is not what you would expect if the methods we developed permitted systematic contamination. More importantly, we also engineer artificial coronas, designed to emulate what we observed in efficacious natural coronas, and these artificial coronas enhance LNP function in vitro and in vivo with striking specificity. If the components used were mere contaminants, then this approach would not work.

Simonsen emphasizes that there is a potential for extracellular vesicle (EV) contamination to affect our results and conclusions. We acknowledge that EVs are known to exhibit a unilamellar morphology, similar to what we observed (Fig. s12^1^), and we are aware of the Minimal Information for Studies of Extracellular Vesicles 2018 (MISEV2018) because we contributed to the development of these guidelines through our co-authorship. There are several reasons why EV contamination is not problematic for our conclusions. Figure s13^1^ demonstrated that the isolated LNPs are relatively monodispersed, unlike EVs which are typically more polydispersed. More importantly, our current work analyzed our samples in depth using proteomics and significant EV contamination should produce signals associated with canonical EV proteins, such as CD9, CD63, CD81, TSG101, Alix (ESCRT-family proteins)^5^, and this is not what we observe (Fig. 3d^1^). Given that EVs can and do interact with PEG^6^, and that we do NOT detect any EV-related proteomic signatures, we can conclude that PEG transfer from LNPs is not sufficient to drive EV pull down in our experiments and/or that EVs do not associate with LNPs. In contrast, proteins that are known to be associated with LNPs, such as ApoE and Vtn^7^, are among the most abundant proteins in our data. Therefore, our experimental evidence does not agree with the speculation that there is significant EV contamination in our samples.

## Particle characteristics

The relationship between plasma and LNP concentrations in terms of corona size is not well studied and the dogma regarding corona formation is based largely on historical data produced (by us among others) using solid lipid nanoparticles (e.g., gold or polystyrene) because these are much easier to handle analytically^8^. Our analysis of LNP corona formation indicates that LNPs are associated with HDL particles. This will make the LNPs larger. Interestingly, similar observations to ours, regarding increases in LNP size of up to 120 nm following corona formation, have recently been reported as well^9^. The LNP size increases we observed (using DLS or NTA analysis) following LNP pull-down are, in Simonsen’s opinion, excessive. We have previously shown that LNP size does indeed increase in the presence of serum^4^, but the sizes we reported in the current work (Fig. s13^1^) were obtained, using unfiltered plasma, following the pull-down procedure, in order to characterize the resulting samples. Furthermore, if the pull-down samples were indeed contaminated by lipoproteins, particularly HDL as speculated by Simonsen, the size measurements would likely exhibit a bias towards smaller values—contrary to our observed results. While accurate in situ size measurements of LNPs with coronas were not the main purpose of this procedure, we do find that the LNPs increase in size following exposure to plasma while remaining functionally and structurally intact (in terms of being able to transfect cells and FRET interactions between the LNP cargo and LNP components).

Simonsen also suggests using Asymmetric Flow Field Flow Fractionation (AF4) as a standard LNP corona isolation control. In addition to being a relatively slow method unsuitable for high-throughput, we are concerned that particles (endogenous and LNPs) and their coronas may be affected by shear forces during the running and focusing steps and prolonged incubation in a buffer environment. In the study that Simonsen cited, the authors also pointed out that AF4 causes the loss of EVs, for example^10^. Although these issues are somewhat resolved through the utilization of fret-inlet channels, we expect a certain amount of shearing and lysis to take place that might affect the coronal content^11^. Once again, the goal of our work was to determine what makes a more effective LNP corona. More detailed characterization of our samples will not change our final conclusions because of the study design, which used in vivo validation of the artificial coronas which we engineered based on our findings.

Finally, our interpretation of the primary NTA data was regarded as problematic by Simonsen. Nanoparticles are difficult to enumerate and sizing is challenging, and we agree that HDL numbers are likely under-estimated by our NTA analysis. This would be problematic if we were attempting to make comparisons between lean and obese phenotypes based on absolute HDL measurements, but these measurements were made to demonstrate that plasma contains particles of similar size to LNPs and, that obese plasma contains more of these particles, as expected, thus validating the phenotype of our animals^12^. In this respect NTA is effective. This does not, however, impact our final conclusions for the reasons already stated repeatedly (we don’t believe PEG-related contamination of our samples is a big problem and the study design negates this problem by using orthogonal validation of our findings).

## Conclusion

LNPs are challenging to process experimentally because they have physical characteristics similar to endogenous particles in the blood and other biofluids. Size, chemical composition, density, spectral characteristics etc., all overlap with endogenous nanoparticles that are also usually much more numerous, and this complicates the analysis. Assay design also has to accommodate throughput, cost and, feasibility.

In the context of our work, we have used therapeutic LNPs containing fluorescent mRNA cargoes to ensure that we are retrieving not just PEG molecules, but also the LNP cargo as well. Using proteomics, we can detect large numbers of proteins, but markers for EVs are notably absent. Furthermore, we see different types of coronas with different LNP compositions, rather than simply pulling down the same serum components consistently as it would be in the case of systematic contamination in the isolation procedure. Finally, the composition of the quantified LNP coronas is different from the parent plasmas. One would assume that if PEGylated lipids were simply transferring to plasma components, then there would be no particular specificity and one should obtain a proteomic pattern containing components from all lipid-related endogenous particles, and this is not what we observe.

We appreciate Simsonsen’s careful evaluation of our work and, we will incorporate more extensive AF4 particle characterization in future experiments. As discussed above, notwithstanding the physiological and chemical similarities among lipoproteins, EVs, and LNPs, significant distinctions persist in their physiochemical properties, origin, formation mechanisms, compositions and, subpopulation heterogeneity. Hence, the adoption of isolation methods previously tailored for disparate nanosized entities necessitates meticulous validation to prevent cross-contaminations and enhance both efficacy and throughput. Finally, with concerns addressed, our opinion is that none of the points raised by Dr. Simonsen alter our conclusions.
